# Temporal atrophy together with verbal encoding impairment is highly predictive for cognitive decline in typical Alzheimer’s dementia – a retrospective follow-up study

**DOI:** 10.3389/fpsyt.2024.1485620

**Published:** 2024-11-19

**Authors:** Burak Doganyigit, Michaela Defrancesco, Timo Schurr, Ruth Steiger, Elke R. Gizewski, Stephanie Mangesius, Malik Galijasevic, Alex Hofer, Noora Tuovinen

**Affiliations:** ^1^ Department of Psychiatry, Psychotherapy, Psychosomatics and Medical Psychology, Division of Psychiatry I, Medical University of Innsbruck, Innsbruck, Austria; ^2^ Department of Radiology, Medical University of Innsbruck, Innsbruck, Austria; ^3^ Neuroimaging Core Facility, Medical University of Innsbruck, Innsbruck, Austria

**Keywords:** Alzheimer's disease, dementia, cognitive assessment, cortical atrophy pattern, magnetic resonance imaging, structural imaging biomarkers

## Abstract

**Introduction:**

The increasing prevalence of Alzheimer’s disease (AD) has created an urgent need for rapid and cost-effective methods to diagnose and monitor people at all stages of the disease. Progressive memory impairment and hippocampal atrophy are key features of the most common so-called typical variant of AD. However, studies evaluating detailed cognitive measures combined with region of interest (ROI)-based imaging markers of progression over the long term in the AD dementia (ADD) stage are rare.

**Method:**

We conducted a retrospective longitudinal follow-up study in patients with mild to moderate ADD (aged 60-92 years). They underwent magnetic resonance imaging (MRI; 3 Tesla, MPRAGE) as well as clinical and neuropsychological examination (Consortium to Establish a Registry for Alzheimer’s Disease [CERAD] -Plus test battery) at baseline and at least one follow-up visit. ROI-based brain structural analysis of baseline MRIs was performed using the Computational Anatomy Toolbox (CAT) 12. Clinical dementia progression (progression index [PI]) was measured by the annual decline in the Mini Mental State Examination (MMSE) scores. MRI, demographic, and neuropsychological data were included in univariate and multiple linear regression models to predict the PI.

**Results:**

104 ADD patients (age 63 to 90 years, 73% female, mean MMSE score 22.63 ± 3.77, mean follow-up 4.27 ± 2.15 years) and 32 age- and gender-matched cognitively intact controls were included. The pattern of gray matter (GM) atrophy and the cognitive profile were consistent with the amnestic/typical variant of ADD in all patients. Deficits in word list learning together with temporal lobe GM atrophy had the highest predictive value for rapid cognitive decline in the multiple linear regression model, accounting for 25.4% of the PI variance.

**Discussion:**

Our results show that temporal atrophy together with deficits in the encoding of verbal material, rather than in immediate or delayed recall, is highly predictive for rapid cognitive decline in patients with mild to moderate amnestic/typical ADD. These findings point to the relevance of combining detailed cognitive and automated structural imaging analyses to predict clinical progression in patients with ADD.

## 1 Introduction

In parallel with an aging population, the prevalence of dementia and the associated economic burden are increasing dramatically, creating an urgent need for rapid and cost-effective methods to diagnose people with neurocognitive disorders and to monitor them in the course of the disease. An estimated 50 million people worldwide have dementia and this number is expected to reach 152.8 million by 2050 (1). Alzheimer’s disease (AD) is the most common cause of dementia accounting for approximately two-thirds of cases in people aged 65 years and older. The current concept of AD postulates a gradual increase in neuropathological changes (ß-amyloid, tau pathology, and cortical atrophy) leading to progressive clinical decline when a critical threshold of these is reached (2, 3). Based on the cognitive profile and atrophy pattern, a distinction is made between typical amnestic and atypical variants of AD dementia (ADD) (e.g., posterior cortical atrophy variant, logopenic variant, primary progressive aphasia, behavioral variant, or dysexecutive variant) (4). The most common typical amnestic variant is characterized by prominent episodic memory impairment and hippocampal atrophy (5). Although there is a relationship between neuropathological changes and clinical symptoms, the time course of cognitive decline and biological disease progression can differ between individuals. Factors such as resilience, cognitive reserve, neuropsychiatric symptoms, white matter pathology, and somatic comorbidities have been linked to a varying dementia progression (6–8). Identifying markers of progression in clinical stages of AD is important for patients, caregivers as well as the health care system to plan appropriate support throughout the course of this still incurable and progressive disease.

Due to lower costs and general availability, magnetic resonance imaging (MRI) as well as clinical and neuropsychological measures are promising markers of ADD progression in clinical routine. However, predicting ADD progression can be challenging as it is a heterogeneous disease with multifactorial interactions rather than a linear longitudinal course (8–10). Neuropsychological variables as markers of ADD progression have yielded inconsistent results depending on the test batteries used and ADD subtypes. The majority of studies report delayed recall of verbal information, rather than encoding, as the most sensitive measure and a key feature of typical ADD (11).

Studies of AD progression typically distinguish between patients with rapid and those with slow progression based on the annual decline in the Mini Mental State Examination (MMSE) score. However, the definition of rapid and slow progression varies between studies, with cut-offs ranging from 2 to 6 points of MMSE score decline per year (12–16). Long-term follow-up studies have found high variability in MMSE score decline across different study populations and numerous factors influencing ADD progression (e.g., age, APO ε status, education, gender, amyloid and tau biomarkers, vascular burden) (8). Therefore, the use of annual MMSE score decline as a continuous variable is a better alternative for the analysis of rapid vs. slow progression with defined cut-offs.

MRI analysis methods have evolved from visual rating assessment of cortical atrophy patterns (17) to automated methods for more precise quantification, e.g., cortical thickness (18, 19) or cortical volume using voxel-based morphometry (VBM) (20). VBM is an MRI-based neuroimaging technique used to quantify differences in gray matter (GM) volumes between groups (21). Large neuroimaging studies on individuals with AD such as the Alzheimer’s Disease Neuroimaging Initiative (ADNI - https://adni.loni.usc.edu/data-samples/adni-data/neuroimaging/mri/) use VBM to quantify GM loss on Magnetization prepared rapid acquisition with gradient echo (MPRAGE) sequence. Automated measures of GM atrophy, particularly of the temporal lobe including the hippocampus, have been proposed as a biomarker of neuronal injury in recent diagnostic criteria for ADD (2, 3).

MRI markers as predictors of disease progression mostly follow the well-established Braak staging model - starting with ß-amyloid, followed by tau pathology in the hippocampus, and finally spreading to other cortical regions (22, 23). In the clinical AD stage, neuropathological changes reach a threshold and clinically symptoms evolve. It is not clear whether the onset of clinical symptoms parallels the current and further atrophy of the brain. Models of different patterns of atrophy and associated distribution of neurofibrillary tangles in AD lead to the definition of three subtypes (typical AD, hippocampal sparing variant, limbic predominant variant) (24). Studies addressing these different subtypes of AD, e.g., the limbic or hippocampal sparing variant, have found varying cognitive and morphological changes over the course of ADD (25, 26). Others who defined GM atrophy factors (temporal, subcortical, cortical) have found a different impact of these atrophy patterns on cognitive decline at different stages of the AD continuum. While temporal atrophy was associated with faster memory decline in Mild Cognitive Impairment (MCI) stage, cortical atrophy in multiple regions was associated with faster cognitive decline in the dementia stage (27).

The aim of this retrospective follow-up study was to evaluate detailed neuropsychological, clinical, and brain atrophy patterns, individually and in combination, as predictors of cognitive decline over time in a sample of patients with ADD. We measured cognitive ADD progression, defined as annual decline in MMSE score, as a continuous rather than a dichotomous variable, by focusing on widely available and cost-effective predictors of dementia progression for clinical and research use to improve care planning and individualized cognitive training for patients with mild and moderate typical/amnestic ADD.

## 2 Materials and methods

### 2.1 Study design

This retrospective observational follow-up study aimed to evaluate the utility of neuropsychological, MRI-based atrophy patterns, and clinical measures as potential predictors of ADD progression. Data of ADD patients who visited the Memory Clinic (Department of Psychiatry, Psychotherapy, Psychosomatics and Medical Psychology) at the Medical University of Innsbruck, Austria, for initial dementia assessment between 2013 and 2019 were retrospectively collected from medical records and MRI scans. Age and gender matched cognitively intact (CI) subjects were matched to the ADD patients as a reference group for brain morphometric analysis. All study participants underwent a 3 Tesla MRI scan and a detailed neuropsychological and clinical examination at baseline. Individuals diagnosed with ADD were continuously monitored, including clinical examination and recording of the MMSE score to assess cognitive decline. CI participants were assessed at baseline and follow-up was recommended in the event of cognitive deterioration, as there was no indication for continuous monitoring. Information on somatic comorbidities (e.g., hypertension, diabetes, hypercholesterolemia), sociodemographic data, and current use of psychotropic and somatic medication was obtained from medical records. The inclusion criteria comprised a diagnosis of mild to moderate probable ADD according to the National Institute on Aging and Alzheimer´s Association (NIA-AA) criteria (28), including i) the presence of subjective memory complaints over the previous 6 months, ii) impaired neuropsychological function of > 2 standard deviations (SD) or more corrected for age and education in one memory function (verbal or figural memory) and at least one other cognitive domain, iii) deficits in activities of daily living assessed with a clinical interview, iv) a Clinical Dementia Rating (CDR) Scale (29) score of ≥ 1 and age ≥ 60 years. Exclusion criteria for ADD patients were severe dementia (MMSE score ≤ 12) or other major mental or neurological disorders (e.g., schizophrenia, Parkinson’s disease). Patients were further excluded if their MRI scans showed strong artifacts, cerebral infarctions, hemorrhage, tumors, hydrocephalus, or severe head trauma or cerebrovascular lesions.

Study participants were classified as “cognitively intact” if they 1) reported mild self-experienced cognitive decline compared to a previous normal state that was unrelated to an acute event or explained by significant psychiatric or somatic disease according to the Subjective Cognitive Decline (SCD) criteria (30) or no self-experienced cognitive decline compared to a previous normal state, and 2) did not fall short of the threshold of 1 SD below the mean of normative data derived from a representative sample in the neuropsychological test battery (CERAD-Plus) and a CDR Scale (29) score of 0.

The final study population comprised 136 participants (104 ADD patients and 32 age- and gender-matched CI controls).

Clinical dementia progression (progression index [PI]) in ADD patients was measured as decline in MMSE scores/time to last follow-up (years) as continuous variable. A lower PI corresponded to a lower average annual decline in MMSE score. To additionally address the concept of defining cognitive decline by setting an MMSE cut-off, we used 2 MMSE points decrease/year as a cut-off based on the rounded median of MMSE declines/year in our study population.

This retrospective study was approved by the Ethics Committee of the Medical University of Innsbruck, Austria (EK Nr: 1046/2018). Due to the retrospective study design, patients and controls did not have to sign an informed consent form.

### 2.2 Clinical and neuropsychological measures

Study participants were tested with a comprehensive neuropsychological battery to assess age- and education-corrected z-scores in several cognitive domains. Different areas of the verbal memory were assessed with the Word List Memory Task as part of the ‘Consortium to Establish a Registry for Alzheimer’s Disease’ (CERAD-Plus) test battery (31). The test included encoding of verbal information (word list learning – three learning trials of 10 words/trial – maximum 30), world list delayed recall (correctly recall of the 10 learned word after a distractor task), word list savings (percentage of recalled word of the last learning trail), and world list recognition (correctly recognized words from word list learning task and correctly classified 10 new words not presented in the leaning task – maximum score 20). Further CERAD verbal fluency (animals/min, s-words/min), object naming (Boston Naming Test [BNT] – maximum score 15), constructional praxis (copying of 4 figures, maximum score 11), figural memory (free recall of the 4 copied figures), and psychomotor speed/mental flexibility (Trail Making Test A and B) were used. The CERAD total score (range 0-100) was calculated as previously described by Chandler et al. (32) and allows an overall judgement of cognitive function based on several subscales. In addition, the CLOX Test part 1 (clock drawing/planning, maximum score 15) (33) and the MMSE (34) were administered. Depression was assessed using the Geriatric Depression Scale – 30 items (GDS) (35). Cerebrovascular burden was assessed by two experienced neuroradiologists (MG, SM) on T2-weighted images using the Fazekas scale (36), a visual four-grade rating scale (0 = no white matter lesions to 3= severe white matter lesions) that quantifies white matter hyperintensities.

### 2.3 MRI acquisition

MRI scans were acquired with either 3 Tesla Siemens Skyra (n=47 in ADD patients and n=14 in CI subjects) or Siemens Verio (n=57 in ADD patients and n=18 in CI subjects). Standardized protocols were applied, including a high-resolution coronal T1-weighted Magnetization Prepared Rapid Gradient Echo (MPRAGE) structural sequence with a voxel size of 0.4 × 0.4 × 1.2 mm (echo time = 2.18 ms, repetition time = 1800 ms, inversion time = 900 ms, flip angle = 9°) and a coronal T2-weighted Fluid Attenuated Inversion Recovery (FLAIR) sequence with a voxel size of 0.7 × 0.7 × 0.3 mm (echo time = 87 ms, repetition time = 8000 ms, flip angle = 150°).

### 2.4 Data pre-processing

A region of interest (ROI) -based brain structural analysis was performed using the Computational Anatomy Toolbox (CAT12). All T1-weighted MRI scans were visually inspected for artifacts and manually oriented around the anterior commissure using Statistical Parametric Mapping (SPM) 12, version 7771 (https://www.fil.ion.ucl.ac.uk/spm/software/spm12/) within MATLAB (version R2022a). The CAT12, version 2170 (https://neuro-jena.github.io/cat/), which is an extension tool in SPM12, was used for further pre-processing steps. These steps comprised bias field correction, segmentation into GM, white matter and cerebrospinal fluid, and registration to the Montreal Neurological Institute (MNI) 152 standard brain template. Registration to MNI space was performed using the Diffeomorphic Anatomical Registration Through Exponentiated Lie Algebra (DARTEL) algorithm provided by CAT12 using a voxel size of 1.0 mm^3^ for normalized images. The resulting GM maps were smoothed with a Gaussian kernel with a half maximum width of 8 mm and were then visually inspected for accuracy of registration. Cortical regional GM volumes of all four lobes (i.e., frontal, temporal, parietal, and occipital), the cerebellum, and subcortical regions were extracted (136 ROIs) according to the computational Neuromorphometrics brain atlas (https://www.neuromorphometrics.com/). While the hippocampal regions are included in the Neuromorphometrics atlas, hippocampal subfields (10 ROIs) provided by the Computational Brain Anatomy (CoBrA) Laboratory atlas (https://www.cobralab.ca/hippocampus-subfields) were included instead to allow a more detailed analysis of these regions, which are considered important to identify for AD pathology.

### 2.5 Statistical analysis

#### 2.5.1 Demographic, clinical, and neuropsychological analyses

To investigate the predictive value of demographic, clinical, and neuropsychological variables on the PI, separate univariate linear regression analyses were performed. Each univariate regression model included one predictor and was adjusted for the baseline covariates of age, gender, MMSE score, and education in years when neuropsychological predictors were included. Unadjusted values are also provided to assess their raw impact on the PI.

Significant (p < 0.2) clinical and neuropsychological predictors of PI were further selected to build multiple linear regression models.

#### 2.5.2 Analysis of morphometric measures

Univariate ROI-wise regression was performed between *a priori* defined ROIs (Neuromorphometrics and CoBrA atlases) of patients’ baseline GM volumes and PI (variable of interest), while additionally including baseline age, gender, MMSE score, years of education and total intracranial volume (TIV) as covariates in CAT12. The threshold for statistical significance was set to an alpha value of 0.05 corrected for multiple comparisons using the false discovery rate (FDR) method. Volumes (ml) of ROIs that significantly predicted the PI were summed up to anatomical scores according to their anatomical location (temporal, frontal, occipital or hippocampal score). For ROIs that were significant in both hemispheres, the score with the higher significance was used. This approach was applied to reduce multicollinearity and increase sensitivity. Prior to anatomical score building, each patient´s ROIs were standardized by their TIV. The internal consistency between ROIs making up the scores was evaluated by calculating Cronbach’s alpha and McDonald’s omega. Principal component analysis (PCA) with oblique rotation was conducted to crosscheck the construct validity of the scores. To analyze the predictive properties of the scores regarding the PI (dependent variable), a linear mixed model was fitted for each summed anatomical score (independent variable) with fixed effects (baseline age, gender, MMSE score, and years of education) and random intercepts for scanner type. Summed anatomical scores showing statistically significant contributions (FDR-corrected *p* < 0.05) to the prediction of the PI were included in a final comprehensive linear model referred to as the ‘MRI-model’. Prior to modeling, scores of each patient were grand mean centered using the scores of the CI group to ease interpretability of the regression model. The brain regional volumes of CI were used to z-transform ADD patient´s regional brain volumes to allow direct comparison between ADD and CI (a low mean z-score in ADD patients reflects great deviation from the CI’s respective mean volume) (**Table 1**).

**Table 1:** Volumetric brain MRI-based regions of interest (ROIs) that significantly predicted the progression index (PI) in 104 Alzheimer’s disease dementia (ADD) patients.

ADD (n=104)			
ROI	Mean z-scores ± SD	t-value	p-value
Left middle temporal gyrus*	-1.207 ± 1.272	4.948	< 0.001
Left inferior temporal gyrus*	-1.124 ± 1.269	3.321	0.015
Left angular gyrus*	-0.964 ± 1.362	3.120	0.018
Left superior temporal gyrus*	-0.560 ± 1.199	2.781	0.027
Left fusiform gyrus*	-1.025 ± 1.144	2.472	0.043
Right middle temporal gyrus	-1.363 ± 1.721	4.272	0.001
Right inferior temporal gyrus	-1.340 ± 1.481	2.825	0.025
Right fusiform gyrus	-1.255 ± 1.395	2.461	0.043
Right angular gyrus	-1.097 ± 1.402	2.449	0.043
**Temporal score**	-1.294 ± 1.337	4.190	< 0.001
Left CA2/CA3*	-1.675 ± 1.726	3.557	0.012
Left CA4/dentate gyrus*	-1.515 ± 1.358	3.098	0.026
Left enthorinal area	-2.119 ± 1.755	2.864	0.025
Left CA1	-1.475 ± 1.466	2.594	0.041
Left amygdala*	-2.565 ± 2.364	2.519	0.043
Right CA2/CA3	-1.225 ± 1.537	2.906	0.026
Right CA4	-1.269 ± 1.463	2.874	0.026
Right amygdala	-2.160 ± 2.343	2.475	0.043
**Hippocampal score**	-2.356 ± 1.911	2.873	0.005
Left medial orbital gyrus	-1.025 ± 1.217	2.456	0.043
Left basal forebrain	-1.284 ± 1.222	3.129	0.018
Left accumbens area	-1.194 ± 1.621	2.425	0.043
Left opercular part of the Inferior frontal gyrus	-0.636 ± 1.068	2.416	0.043
Left superior frontal gyrus medial segment	-1.522 ± 1.496	2.405	0.043
Left frontal pole	-0.558 ± 1.330	2.400	0.043
Right basal forebrain*	-1.169 ± 1.246	3.719	0.005
Right triangular part of the inferior frontal gyrus	-0.795 ± 0.890	2.855	0.025
**Frontal score**	-1.459 ± 1.464	3.234	0.002
Left lingual gyrus	-0.698 ± 1.198	3.761	0.005
Left inferior occipital gyrus	-0.357 ± 1.213	2.833	0.025
Left cuneus	-0.686 ± 1.139	2.826	0.025
Left middle occipital gyrus	-0.354 ± 0.943	2.999	0.023
**Occipital score**	-0.684 ± 1.118	-4.033	0.001

Z-scores (z-transformed using regional brain volumes of cognitively intact group) are shown but analysis was carried out with volumes (ml). ROI-wise regression was controlled for baseline age, gender, Mini Mental State Examination (MMSE) score, years of education, total intracranial volume (TIV), and scanner type* The side with the smaller p-value was considered for analyses.

CA, Cornu Ammonis.

#### 2.5.3 Analyses of combined factor model

Predictors of different models (neuropsychological and MRI model) were combined in a single multiple linear regression to determine their collective predictive utility towards the PI. Predictors were entered in a stepwise forward manner to the model, which was adjusted for baseline age, gender, MMSE score, and years of education. At each iteration, one predictor was added to the model and only kept permanently if it led to significant improvements in terms of the Bayesian information criterion (BIC). The predictors were added to the model based on the ascending order of their p-values in the initial models (neuropsychological and MRI model).

## 3 Results

### 3.1 Demographic and clinical characteristics

We analyzed data of 104 ADD patients (aged 63 to 90 years, mean age of 79.13 ± 5.81 years, 73% female) and 32 age- and gender-matched CI controls (mean age of 77.72 ± 5.80 years, 72% female).

Detailed demographic and clinical characteristics of the 104 ADD patients and their predictive values on the PI are presented in **Table 2**. Unadjusted values are also provided to assess their raw impact on the PI. Demographic and clinical characteristics of CI controls used for grand mean centering and z-transformation of regional morphometric measures are presented in Appendix 3.

**Table 2:** Demographic and clinical characteristics of 104 Alzheimer’s disease dementia (ADD) patients and their predictive value on the progression index (PI) in univariate linear regression.

ADD (n=104)				
Demographic variables	Mean ± SD or N (%)	Adjusted β [unad.]	Adjusted SE [unad.]	Adjusted p [unad.]
Age (years)	79.13 ± 5.81	-0.182 [-0.191]	0.098 [0.097]	0.066 [0.053]
Female (%)	76 (73.1)	0.037 [-0.086]	0.220 [0.222]	0.867 [0.698]
Education (years)	10.03 ± 2.36	0.168 [0.127]	0.099 [0.098]	0.092 [0.200]
Clinical variables				
Progression index^1^	1.66 ± 1.77	—	—	*—*
Follow-up duration (years)	4.27 ± 2.15	—	—	—
MMSE raw score at baseline	22.66 ± 3.69	-0.210 [-0.229]	0.097 [0.096]	0.032 [0.019]
MMSE raw score at follow-up	16.26 ± 7.23	—	—	—
GDS- 30 items (raw score)	8.04 ± 5.70	-0.053 [-0.074]	0.098 [0.099]	0.586 [0.456]
Fazekas score	1.42 ± 0.85	0.070 [0.102]	0.102[0.099]	0.497 [0.800]
Hypertension	61 (58.7)	-0.055 [-0.189]	0.203 [0.199]	0.789 [0.347]
Diabetes^2^	10 (9.6)	—	—	—
Hypercholesterolemia	42 (40.4)	-0.104 [-0.138]	0.197 [0.200]	0.600 [0.492]
APO ε (% ε4-carriers)	52 (50.0)	-0.042 [0.010]	0.216 [0.214]	0.845 [0.643]

^1^decline of MMSE scores/time to last follow-up, ^2^only descriptive statistics provided due to small prevalence. Regression models were controlled for baseline age, gender, MMSE score, and years of education.

MMSE, Mini Mental State Examination; GDS, Geriatric Depression Scale; SE, standard error.

Age, PI, and baseline MMSE scores of ADD patients were balanced between genders, whereas the educational level was significantly higher in males (Z = 2.665, p = 0.008). Mean MMSE scores of patients decreased from 22.66 ± 3.69 at baseline (range 13 to 29, 25% ≥ 20 points) to 16.26 ± 7.23 at follow-up (range 3-28). Results of univariate regression analysis with demographic and clinical variables as predictors and the PI as dependent variable showed that a lower MMSE score at baseline significantly predicted rapid cognitive decline (β = -0.210, p = 0.032). Mean follow-up time was 4.27 ± 2.15 years (range 1.07 to 9.78 years). Hypertension (59%) was the most frequent cardiovascular comorbidity, followed by hypercholesterolemia (40%) and diabetes (10%). No other demographic or clinical variable reached statistical significance (p < 0.05).

### 3.2 Association between neuropsychological test performance and cognitive decline (neuropsychological model)

Detailed results of the univariate linear regression model for neuropsychological test performance are presented in **Table 3**.

**Table 3:** Neuropsychological test performance of 104 Alzheimer’s disease dementia (ADD) patients and its predictive value for the progression index (PI) in univariate linear regression.

ADD (n=104)				
Neuropsychological variables	Mean ± SD	β	SE	*p*-value
Constructional praxis^†^	-0.49 ± 1.42	0.179	0.100	0.078
Constructional recall^†^	-1.97 ± 0.90	-0.056	0.101	0.583
BNT^†^	-0.60 ± 1.46	-0.160	0.010	0.111
Word list learning^†^	-2.70 ± 1.63	-0.424	0.102	< 0.001
Word list delayed recall^†^	-2.29 ± 1.04	-0.199	0.101	0.052
Word list savings^†^	-1.73 ± 1.51	-0.055	0.095	0.565
Word list recognition^†^	-2.07 ± 1.29	-0.040	0.107	0.710
Verbal fluency (animals)^†^	-1.44 ± 1.02	-0.109	0.106	0.306
Verbal fluency (s-words)^†^	-0.70 ± 1.14	0.051	0.110	0.645
Trail Making Test A^†^	-1.61 ± 1.18	-0.030	0.096	0.760
Trail Making Test B^a^	-1.10 ± 0.98	—	—	—
CLOX I (raw total score)	9.46 ± 3.51	-0.090	0.100	0.366
CERAD total score (raw total score)	52.01 ± 11.14	-0.327	0.123	0.009

a only descriptive statistics provided due to 6 missings, † Baseline z-scores are displayed, but raw scores were used in regression analysis. Regression models were controlled for baseline age, gender, Mini Mental State Examination (MMSE) score, and years of education.

BNT, Boston Naming Test; CERAD, Consortium to Establish a Registry for Alzheimer’s disease-Plus; SE, standard error.

Results of univariate regression with neuropsychological variables (independent variables) and the PI (dependent variable) showed that lower baseline scores in word list learning (β = -0.424, p < 0.001*)* and the CERAD total score (β = -0.327, p = 0.009) predicted higher PI.

Based on the univariate linear regression analyses, predictors with p-values < 0.2 (i.e., CERAD total score, BNT, word list learning, word list delayed recall, and constructional praxis) were simultaneously entered into a multiple linear regression model (neuropsychological model) with the PI as the dependent variable. Control variables in the model were baseline age, gender, MMSE score, and years of education. Three patients were excluded due to missing data. Detailed results of the multiple linear regression model are presented in **Table 4**. The model was significant (F[9,91] = 3.63, p < 0.001) and explained 19.1% of PI variance (R²). The only predictor that reached significance was word list learning (β = -0.485, p = 0.011).

**Table 4:** Multiple linear regression model (neuropsychological model) with neuropsychological variables as independent variables and the progression index (PI) as dependent variable in 101 Alzheimer´s disease dementia (ADD) patients.

ADD (n=101)					
		β	SE	t-value	p-value
Predictors	CERAD total score	0.188	0.233	0.805	0.423
	BNT	-0.167	0.116	-1.435	0.155
	Word list delayed recall	-0.081	0.116	-0.694	0.490
	Word list learning	-0.485	0.186	-2.602	0.011
	Constructional praxis	0.098	0.109	0.892	0.375
Model	F	R²	adj. R²	BIC	p-value
	3.630	0.264	0.191	305.40	0.001

Model was controlled for baseline age, gender, Mini Mental State Examination (MMSE) score, and years of education. Raw scores were used.

CERAD, Consortium to Establish a Registry for Alzheimer’s disease-Plus; BNT, Boston Naming Test; BIC, Bayesian information criterion; SE, standard error.

Additional analyses comparing neuropsychological test performance in patients with rapid and slow progression using a 2-point cut-off for annual decline in MMSE score (PI) revealed significantly lower baseline values in terms of the CERAD total score (Z = -2.546, p = 0.011), word list learning (Z = -3.529, p < 0.001), word list saving (Z = -2.577, p = 0.010), and constructional praxis (Z = -2.093, p = 0.036) in the rapid progression group (N= 40, 38.5%).

### 3.3 Brain morphometry and cognitive decline: results of regression analysis (MRI model)

**Figure 1:**
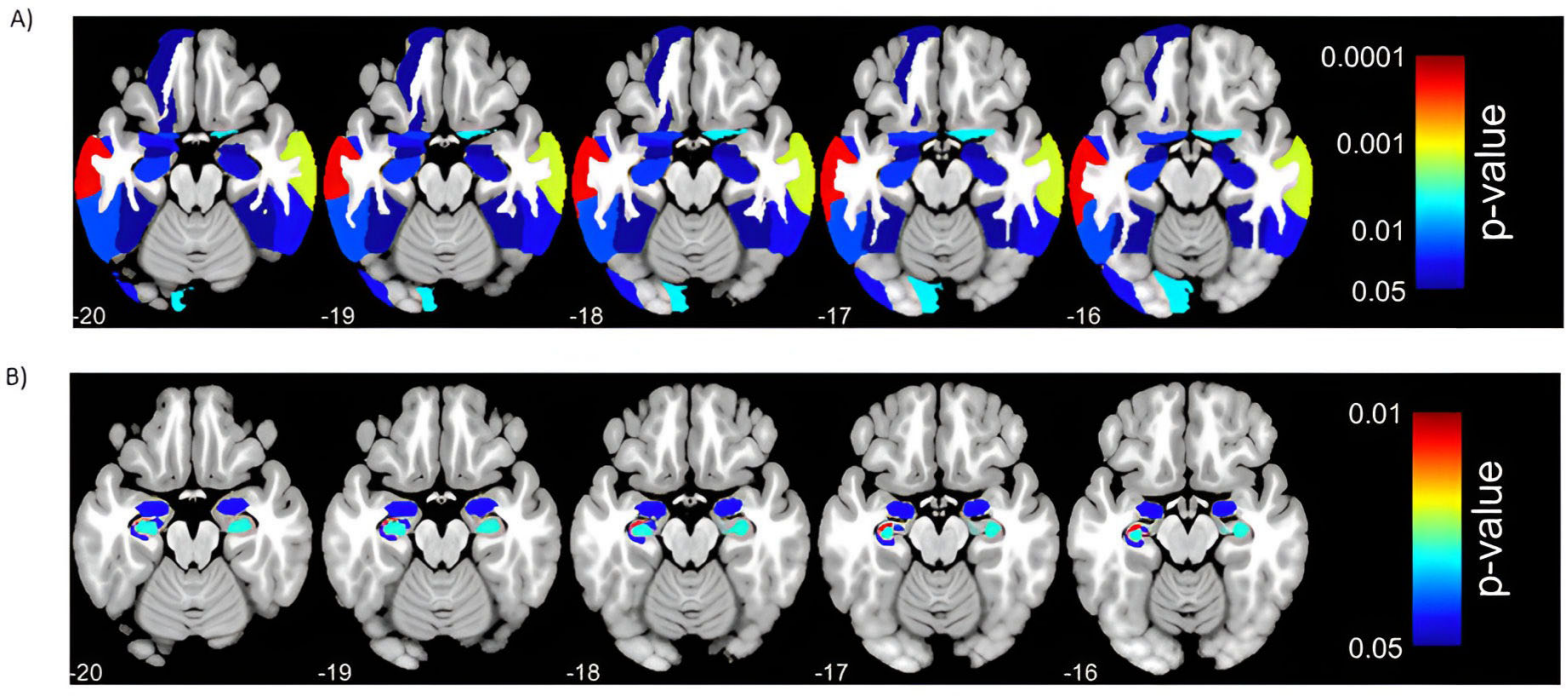
Significant (p < 0.05, FDR corrected) results of univariate regression analysis between regions of interest (ROIs) (independent variables)and the progression index (PI, dependent variable) in Alzheimer’s disease dementia (ADD) patients. Panel **A** depicts associations between ROIs from the Neuromorphometrics Atlas and PI. Panel **B** depicts associations between hippocampal subfield ROIs from the CoBra Atlas and PI. Models were controlled for baseline age, gender, Mini Mental State Examination (MMSE) score, years of education, scanner type, and total intracranial volume (TIV).

Univariate ROI-wise regression analysis revealed that GM volumes of 29 ROIs significantly (FDR corrected *p*-value < 0.05, z-transformed, standardized by CI controls) predicted the PI in ADD patients (**Table 1**, **Figure 1**). The four sum scores (temporal, frontal, occipital, hippocampal score) were formed from 21 selected ROIs (excluding 8 ROIs that occurred in both left and right hemispheres). ROIs of hippocampal regions showed the most pronounced GM atrophy with a sum score below -2 SDs, whereas GM volumes of occipital regions were within the normal range. The left angular gyrus was the only parietal ROI selected and is included in the temporal score, as suggested by a PCA performed on selected ROIs (see Appendix 1). No other of the 12 included parietal ROIs predicted the PI and had a z-score below -1.

All mixed linear model analyses with scanner type as a random intercept and summed anatomical scores as fixed effects were significant (p-value < 0.05), indicating that lower sum scores of the four anatomical regions separately significantly predicted higher PI (**Table 5**). The lowest BIC (324.987) was observed for the model with the temporal sum score as a predictor. Scanner type as a random intercept showed little variance between summed anatomical scores and was excluded from further analyses as a confounding variable.

**Table 5:** Linear mixed models for summed anatomical scores (i.e., temporal, frontal, occipital, hippocampal score) with random intercepts for scanner type and progression index (PI) as dependent variable in 104 Alzheimer’s disease dementia (ADD) patients.

ADD (n=104)				
Parameter	Hippocampal score	Temporal score	Frontal score	Occipital score
Random Effects				
Scanner type intercept (SD)	2.57 × 10^-5^	3.20 × 10^-5^	3.76 × 10^-5^	3.97 × 10^-5^
Residuals (SD)	0.932	0.895	0.925	0.901
Fixed Effects				
Score coefficient (β)	-0.280	-0.429	-0.318	-0.365
SE	0.097	0.103	0.100	0.091
p-value	0.005	< 0.001	0.002	< 0.001
BIC	326.961	324.987	333.459	326.475

Each model was adjusted for baseline age, gender, Mini Mental State Examination (MMSE) score, and years of education. All models comprise a single region of interest (ROI)-analysis derived summed anatomical score (hippocampal, temporal, frontal, or occipital) as independent variable. Each patients ROI-measures were standardized by total intracranial volume (TIV) prior to score building. Scores were grand mean centered by means of cognitively intact (CI) subjects’ respective scores. P < 0.05 was considered significant.

SD, standard deviation; SE, standard error; BIC, Bayesian information criterion.

When the four summed anatomical scores were simultaneously added as predictors in a multiple linear model with the PI as the dependent variable, the model was significant (F[8,95] = 4.572, p < 0.001), explaining 21.7% of the variance (R²). None of the four summed anatomical scores as predictors reached significance (see Appendix 2).

### 3.4 Combined impact of brain morphometry and neuropsychological test performance on cognitive decline

Results of the multiple linear regression model combining MRI and neuropsychological data to predict PI are presented in **Table 6**. Inclusion of word list learning in the regression model accounted for 19.7% of PI variance (R²) (F[5,95] = 5.907, p < 0.001). Adding the temporal score to the model significantly increased R² to 25.4% (F[6,94] = 6.675, *p* < 0.005) and decreased the BIC from 290.584 to 286.697. Adding any other remaining predictor from the MRI or the neuropsychological model did not improve model fit in terms of BIC and did not explain any further variance. Predictors and models remained significant (*p* < 0.05) throughout the iterative process.

**Table 6:** Multiple linear regression model combining neuroimaging and neuropsychological variables to predict progression index (PI) in 101 Alzheimer’s disease dementia (ADD) patients.

ADD (n=101)					
		β	SE	t-value	p-value
Predictors	Word list learning	-0.361	0.111	-3.252	0.002
	Temporal score	-0.307	0.107	-2.873	0.005
Final model	F	R²	adj. R²	BIC	p-value
	6.675	0.295	0.254	286.697	0.001

Model was controlled for baseline age, gender, MMSE scoe, years of education, and total intracranial volume (TIV). Temporal score was grand mean centered by means of cognitively Intact (CI) subjects’ respective scores. P < 0.05 was considered significant.

SE, standard error; BIC, Bayesian information criterion.

## 4 Discussion

This study analyzed the role of automated MRI-based ROI measurements of the brain and neuropsychological assessments, separately and in combination, as predictors of cognitive decline in patients with mild to moderate typical ADD over up to 10 years. Baseline cortical atrophy was most pronounced in the hippocampus, followed by atrophy in frontal, temporal, and less so in occipital regions. Neuropsychological examination revealed significant cognitive deficits in verbal and figural memory, with the strongest impairment in the encoding of verbal information.

Lower baseline performance in word list learning together with temporal GM atrophy (temporal score) was most predictive of rapid clinical dementia progression, measured as annual decline in MMSE score (PI) in a multiple regression model. The combination of neuropsychological and morphometric variables exceeded the significance of each modality alone.

### 4.1 Impact of neuropsychological and clinical measures on cognitive decline

We investigated an older population with mostly (75%) mild dementia and no to mild cerebrovascular burden. Notably, gender did not impact cognitive decline. This result is in line with a comparable study by Özge et al. (6), although others have found contradictory results (37). Clinical dementia progression, as measured by the annual decline in MMSE score (PI), showed an average mild progression with a mean PI of 1.7 over approximately 4 years of follow-up.

Baseline neuropsychological testing, including verbal and figural memory, executive function, and language-related functions, revealed the most pronounced cognitive deficits in memory functions. Therefore, our study population can be classified as having the most common variant known as typical/amnestic ADD. Our finding of relatively mild cognitive decline in an older study population with predominant memory impairment is consistent with previous studies reporting more rapid dementia progression in atypical AD and in people of younger age (25). Similarly, a study by Musicco et al. (38) found slower dementia progression, as measured by MMSE decline, in late-onset AD. However, that study did not provide data on AD variants or morphological data (38).

A lower MMSE score at baseline was associated with faster cognitive decline, regardless of whether the dementia was mild or moderate (MMSE cut-off: 20 points). Our findings are consistent with previous studies reporting that a lower baseline MMSE score is associated with a more rapid cognitive decline in the course of AD dementia (39), (40). Although the MMSE is a very crude instrument, its validity for assessing global cognitive function and clinical dementia progression has been reported in numerous studies (41). For this reason, most studies that examine patients from mild to severe stages of dementia use the MMSE as a progression parameter. However, the MMSE should not be used as the only predictor of conversion from MCI to AD dementia due to its low specificity in early clinical stages of ADD (42). Although we suggest that more extensive measurements of different cognitive domains up to the severe stages of AD dementia would be useful, the increasing deficits in attention, concentration and perception in the course of AD preclude such longitudinal assessments. Therefore, long-term follow-up studies of patients from mild to severe stages of ADD must use different measures of disease progression compared to studies of MCI to mild ADD. As in this study, the combined use of comprehensive baseline cognitive and clinical measures as predictor variables and the MMSE score as a continuous measure of disease progression over the course of ADD can be considered as an appropriate study design.

According to univariate analysis, we found that poorer performance in word list learning and a lower CERAD total score at baseline predicted rapid cognitive decline when controlling for age, gender, years of education, and ADD severity (MMSE score). However, the multiple regression model (neuropsychological model) was significant and explained 19.1% of the variance, with word list learning being the only predictor to make a significant unique contribution. This finding was somewhat unexpected given that the current literature and diagnostic criteria include primary storage but not encoding impairment in typical ADD (43). Studies of cognitive markers of AD progression have also identified verbal memory impairment as a relevant factor for clinical worsening, at least in some subtypes of AD (8, 26). However, most studies that analyze multiple neuropsychological variables in detail focus on clinical progression in the MCI stage of AD rather than the dementia stage (44). Furthermore, a more precise subdivision of the different areas of verbal memory (e.g., encoding, delayed and immediate recall) is often lacking. We hypothesize that this distinction is important for patients in the advanced stages of dementia. Although AD can be viewed as a continuum, the onset of clinically significant symptoms may mark a critical change in the course of the disease and be a clear sign that a threshold of cognitive and biological disease markers has been crossed. We believe that beyond these critical thresholds, other factors influencing dementia progression (e.g., somatic comorbidities) are reliable compared to early and preclinical stages of AD.

Previous studies have suggested that encoding deficits in ADD result from reduced attention or lack of recall strategies. Accordingly, encoding impairment could be a specific progression marker for ADD patients with significant attentional deficits. Attention deficits, in turn, are associated with changes in many brain areas and are likely related to advanced global cortical atrophy. It is also important to note that a valid delayed recall test depends on good encoding skills and that a floor effect of delayed recall tests must be considered in advanced stages of dementia (11). Consistent with these considerations, a study by Xie et al. found no predictive value of repeated delayed recall tests in ADD patients over a follow-up period of up to 100 months (45). However, that study did not report word list learning outcomes, but the data presented in the publication suggest a floor effect of delayed recall measures over time.

It is somewhat surprising that we did not find impairment in non-amnestic cognitive domains as a predictor of cognitive decline, even though frontal atrophy was the second most prominent GM atrophy pattern. However, using the concept of defining cognitive decline by setting an MMSE cut-off of 2 points/year was associated with more deficits in verbal memory (encoding and delayed recall of learned information) and visuoconstructive abilities in the group with more than 2 points decline per year. Frontal lobe atrophy may be responsible for the latter. Our findings may be explained by the fact that most of the measures included in the model are associated with memory, attention, and language functions and involve multiple interconnected cortical areas. In support of this, the CERAD total score, as sum of neuropsychological measures, was a significant predictor of cognitive decline in univariate but not in multiple regression analyses. This supports the significant predictive value of verbal memory impairment in typical ADD patients, as non-amnestic variables did not predict the PI despite the fact that multicollinearity was ruled out by checking the variance inflation factor (VIF < 5).

### 4.2 Measures of brain atrophy as a marker of cognitive decline

We performed ROI analyses of GM volumes in different cortical and subcortical regions to assess the predictive value of cortical atrophy for clinical dementia progression. To rule out known factors influencing GM volumes (e.g., hippocampal GM atrophy) (46), the data were corrected for age, gender, TIV, and MMSE score at the time of MRI. We found that left-dominant hippocampal atrophy, including atrophy of the amygdala, was particularly evident given the extent of cortical atrophy. Most hippocampal subfields yielded a GM volume with a z-score below minus 2 SDs, corresponding to significant volume loss. Our findings are in line with the current literature, which consistently describes hippocampal atrophy as a key feature of AD. Accordingly, early neuropathological and VBM studies found GM atrophy of the hippocampus and the temporal lobe in patients with mild-to-moderate stages of ADD (47, 48).

Most studies have found hippocampal and amygdalar atrophy as a marker of early clinical AD stage (49–51) but not as a marker of clinical or neuropathological disease progression in dementia stage. For example, Risacher et al. examined the clinical course of disease in different variants of ADD in a 2-year follow-up study. Clinical progression in amnestic ADD patients with typical hippocampal atrophy at baseline was mostly associated with global rather than hippocampal atrophy (25). In some respects, we found that atrophy of the other brain regions studied (temporal, occipital, and frontal lobes) predicted rapid cognitive decline when analyzed separately. However, temporal lobe atrophy was the most predictive of further rapid cognitive decline. Our MRI findings support the Braak staging concept of AD which describes an upstream progression of neuropathological brain changes, such as neurofibrillary tangle formation starting along the hippocampal tract and later spreading to the temporal, parietal, and finally frontal neocortices (22). Fittingly, we found the most pronounced volume loss in the hippocampus and the amygdala, corresponding to Braak stage I-III, followed by atrophy in the temporal lobe (Braak stage IV-V), less pronounced in the frontal and occipital lobes, and no significant GM loss in the parietal regions. Based on Braak’s concept, it can be assumed that GM atrophy of different brain regions corresponds to the AD stage, the AD variant, and the disease progression.

### 4.3 Combined neurocognitive and atrophy measures to predict cognitive decline

Although impaired verbal memory and the CERAD total score as well as atrophy scores of cortical regions were individually predictive of rapid cognitive decline, the combination of neuropsychological and structural variables exceeded the significance of each modality alone. In contrast to the typical cognitive and imaging features of amnestic/typical ADD, we found that impaired word list learning, but not delayed recall, and temporal atrophy, but not hippocampal atrophy, predicted rapid cognitive decline. Nevertheless, numerous studies on humans and animal models provide evidence for a close association of the hippocampus and the temporal lobe with the ability to learn and recall verbal information (52–54). Our finding of encoding impairment as a cognitive predictor of rapid MMSE decline is therefore consistent with the measured pattern of GM atrophy in our study population. Importantly, markers of progression in the early and preclinical stages of AD need to be distinguished from those in the clinical AD stages. Studies assessing detailed markers of ADD progression over time are rare and, at least to our knowledge, none have focused on amnestic/typical ADD. In some respects, our results are consistent with those of Lenhart et al. who reported distinct atrophy subtypes, including the hippocampal formation, that were associated with verbal memory impairment in a 1-year follow-up study of probable AD patients (26). However, the follow-up period was short compared to our study, which means that the information on dementia progression is limited. In summary, we agree with the conclusion of a review on the prediction of the longitudinal course of dementia by Melis et al. which reports a large heterogeneity in studies addressing markers of dementia progression (9). However, based on our findings and previous studies, we recommend a combined detailed assessment of clinical, cognitive, and structural markers for prospective follow-up studies in ADD patients to increase their validity for clinical routine.

### 4.4 Limitations

As our final study population consisted exclusively of patients with typical/amnestic ADD, we were unable to perform within-group regression analyses to examine the effects of clinical phenotype (atypical non-amnestic vs. typical amnestic ADD). We hypothesize that the exclusive consideration of this phenotype is related to our restrictive exclusion criteria, which include the presence of significant neuropsychiatric symptoms, somatic comorbidities, and cerebrovascular burden, which are common in atypical variants of ADD. However, to our knowledge, no other study has examined the detailed clinical, imaging, and cognitive markers of long-term progression in typical/amnestic ADD patients. Additionally, it is important to note that our study population comprised Caucasians only, with no individuals of African, Hispanic, Latino, or Asian background. Consequently, the generalizability of our results to other ethnicities is limited. The different observation periods, ranging from one to ten years, may further limit the generalizability of our findings. As we do not have detailed information on non-drug treatments (e.g., cognitive training) received by participants during the study period, we cannot rule out the possibility that such treatments may have influenced cognitive decline. Based on the clinical, imaging (temporal and particularly GM atrophy of the hippocampus), and cognitive profile at baseline and cognitive decline at follow-up, the diagnosis of typical ADD is highly likely, although other biomarkers (amyloid and tau) were not evaluated to further confirm the diagnosis. Finally, the choice of ROIs was data driven and based on the current literature, comparability with other structural imaging analyses (e.g., FMRIB Software Library [FSL] or FreeSurfer based analyses) is limited. Although the small sample size can be seen as a limitation, the statistical methods used enabled us to achieve highly significant and robust results.

### 4.5 Conclusion

Our findings support the concept of a typical/amnestic variant of ADD with a predominant temporal atrophy pattern. In contrast to previous studies, we were able to demonstrate that impairment in the encoding of verbal material, rather than in delayed recall, is highly predictive of rapid cognitive decline in patients with mild to moderate ADD. We also found that GM atrophy of the hippocampus is most pronounced in the typical/amnestic variant of ADD, but that temporal atrophy is critical for the rapid progression of symptoms. Finally, our results support the usefulness of a combined use of detailed cognitive and automated structural imaging techniques to predict cognitive decline in ADD patients. We anticipate that our findings may help guide treatment planning and individualized cognitive training focused on improving memory and attention in patients with typical/amnestic ADD in clinical practice or research settings.

## Data Availability

The raw data supporting the conclusions of this article will be made available by the authors, without undue reservation.
