# Supplementary material for: Temporal atrophy together with verbal encoding impairment is highly predictive for cognitive decline in typical Alzheimer’s dementia – a retrospective follow-up study
Source: Front Psychiatry. 2024 Nov 19;15:1485620. doi: 10.3389/fpsyt.2024.1485620 (PMC11611803; doi:10.3389/fpsyt.2024.1485620)
Supplement: Supplementary file 1 [file Table1.docx]

Supplement 1: Four components extracted by principal component analysis (PCA) applied on volumes of 21 regions of interest (ROI) of Alzheimer’s disease dementia (ADD) patients (N = 104).

|  |  |  |  |  |
| --- | --- | --- | --- | --- |
|  | Components | | | |
| **ROI** | **Hippocampal** | **Frontal** | **Temporal** | **Occipital** |
| Left CA4 | 0.94 |  |  |  |
| Left CA1 | 0.91 |  |  |  |
| Left CA2/CA3 | 0.86 |  |  |  |
| Left amygdala | 0.81 |  |  |  |
| Left enthorinal area | 0.76 |  |  |  |
| Left frontal pole |  | 0.80 |  |  |
| Right triangular part of the inferior frontal gyrus |  | 0.76 |  |  |
| Left superior frontal gyrus medial segment |  | 0.73 |  |  |
| Left opercular part of the inferior frontal gyrus |  | 0.69 |  |  |
| Left medial orbital gyrus |  | 0.67 |  |  |
| Left accumbens area |  | 0.62 |  |  |
| Right basal forebrain |  | 0.47 |  |  |
| Left angular gyrus |  |  | 0.72 |  |
| Left superior temporal gyrus |  |  | 0.72 |  |
| Left middle temporal gyrus |  |  | 0.71 |  |
| Left inferior temporal gyrus |  |  | 0.63 |  |
| Left fusiform gyrus |  |  | 0.62 |  |
| Left middle occipital gyrus |  |  | 0.60 | 0.45 |
| Left lingual gyrus |  |  |  | 0.77 |
| Left inferior occipital gyurs |  |  |  | 0.53 |
| Left cuneus |  |  |  | 0.77 |

Note. Principal component analysis (PCA) was performed with oblique rotation. Only loadings 0.40 ≥ are shown. Volumes were standardized by total intracranial volumes (TIV) and z-transformed by means of cognitively intacts (CI) subjects' respective volumes. Abbreviations: Cornus Ammonis (CA).
