# Supplementary material for: Temporal atrophy together with verbal encoding impairment is highly predictive for cognitive decline in typical Alzheimer’s dementia – a retrospective follow-up study
Source: Front Psychiatry. 2024 Nov 19;15:1485620. doi: 10.3389/fpsyt.2024.1485620 (PMC11611803; doi:10.3389/fpsyt.2024.1485620)
Supplement: Supplementary file 2 [file Table2.docx]

Supplement 2: Multiple linear regression model with four region of interest (ROI) derived summed anatomical scores (hippocampal, temporal, frontal, occipital) as independent variables and the progression index (PI) as dependent variable in Alzheimer's disease dementia (ADD) patients (N = 104).

|  |  |  |  |  |  |
| --- | --- | --- | --- | --- | --- |
| ADD (n=104) | | | | | |
|  |  | β | SE | t-value | p-value |
| Predictors | Hippocampal score | -0.085 | 0.110 | 0.777 | 0.439 |
|  | Temporal score | -0.162 | 0.161 | -1.008 | 0.316 |
|  | Frontal score | -0.118 | 0.122 | -0.971 | 0.334 |
|  | Occipital score | -0.210 | 0.112 | -1.871 | 0.064 |
| Model | F | R² | adj. R² | BIC | p-value |
|  | 4.572 | 0.278 | 0.217 | 306.704 | <.001 |

Note. Model was adjusted for age, sex, baseline Mini Mental State Examination (MMSE) scores, years of education and total intracranial volume (TIV). Scores were grand mean centered by means of cognitively intact (CI) subjects' respective scores. P < .05 was considered significant.

Abbreviations: standard error (SE), Bayesian information criterion (BIC).
