# Supplementary material for: Temporal atrophy together with verbal encoding impairment is highly predictive for cognitive decline in typical Alzheimer’s dementia – a retrospective follow-up study
Source: Front Psychiatry. 2024 Nov 19;15:1485620. doi: 10.3389/fpsyt.2024.1485620 (PMC11611803; doi:10.3389/fpsyt.2024.1485620)
Supplement: Supplementary file 3 [file Table3.docx]

Appendix 3: Demographic and clinical characteristics of cognitive intact (CI) participants

| CI (n=32) | |
| --- | --- |
| Demographic variables | Mean ± SD or N (%) |
| Age (years) | 77.59 ± 5.86 |
| Female (%) | 23 (71.88) |
| Education (years) | 11.06 ± 2.44 |
| Clinical variables |  |
| MMSE at baseline (raw score) | 28.56 ± 1.13 |
| GDS- 30 items (raw score) | 7.28 ± 4.61 |
| Fazekas score | 1.19 ± 0.64 |
| APO ɛ (% ɛ4-carriers) | 7 (22%) |

Abbreviations: Mini Mental State Examination (MMSE),

Geriatric Depression Scale (GDS), Cognitive Intact (CI)
